# Combination of Sea Sand Disruption Method and Ion-Pair Solid-Phase Extraction for Effective Isolation and Purification of Chlorogenic Acid from Plants Prior to the HPLC Determination

**DOI:** 10.3390/molecules27175601

**Published:** 2022-08-31

**Authors:** Dorota Wianowska

**Affiliations:** Department of Chromatography, Institute of Chemical Sciences, Faculty of Chemistry, Maria Curie-Skłodowska University in Lublin, Pl. Maria Curie-Skłodowska 3, 20-031 Lublin, Poland; dorota.wianowska@poczta.umcs.lublin.pl

**Keywords:** sea sand disruption method, ion-pair solid-phase extraction, CQA purification, fractionation of plant extracts, sample preparation

## Abstract

Chlorogenic acid (CQA) is one of phenolics commonly found in higher plants, possessing numerous health-promoting effects on humans. Unfortunately, it is easily degraded/transformed into other substances during extraction. Therefore, its reliable analysis requires a special approach that does not involve high temperatures. This paper presents a very simple method of CQA isolation using the sea sand disruption method with subsequent purification of the extract using the ion-pair solid-phase extraction process, followed by HPLC–DAD detection. It was found that control of the ion pairing reagent concentration and sample pH is crucial to improve purification, and that the best results, with recovery exceeding 98%, were obtained for 0.05 M tetrabutylammonium bisulfate at pH 7 when the ion pairs were formed directly in the extract and eluted from the C18 sorbent using an acidified methanol–water mixture. The practical potential of the developed procedure was verified by using it for CQA isolation from different plants. The approach represents one of the contemporary analytical trends and current advances in the solid phase extraction, in which several sorption extraction techniques are combined to ensure high-quality analytical results.

## 1. Introduction

Chlorogenic acid (CQA), the monoester of caffeic acid with quinic acid, is the most abundant phenolic acid in higher plants [1]. Its pro-health properties, including anti-obesity, anti-inflammatory, analgesic, antipyretic, antimutagenic, antiviral and anti-tumorous ones, are well known [2,3,4]. This compound is also believed to be the most active antioxidant component [5] and as such it can promote the prevention of type 2 diabetes and cardiovascular diseases [6]. Hence, there is great interest in developing new methods for its extraction from plants and reliable analysis, all the more given that CQA is widely used as a quality marker in the control of various phytochemical plants and drugs [7,8].

The analytical procedures for determining plant constituents involve the application of a sample preparation method to fully isolate and/or preconcentrate the analysed substances from the plant matrix prior to chromatographic analysis. Most plant analysis procedures rely on high-temperature liquid extraction methods, such as extraction via a Soxhlet apparatus and extraction under reflux. For several years researchers have been focused on so-called enhanced extraction techniques that enable the full recovery of compounds of interest from the sample matrix in a short time. One of these is pressurized liquid extraction (PLE), recognized as a leading method for efficient and quick sample preparation, especially in plant analysis. However, at high temperatures CQA is degraded and transformed into other compounds [9,10,11,12], and even a quick extraction using the PLE technique is not able to eliminate this process [13]. Therefore, a different approach for CQA isolation from plant materials is proposed. In [14] it was shown that the sea sand disruption method (SSDM), also known as the matrix solid-phase dispersion (MSPD) method, is the most appropriate technique for the estimation of the actual CQA content in plants. This is a simpler version of the solid-phase extraction (SPE) technique and generally consists in homogenization of a plant sample with a solid abrasive material (sand in this case) and subsequent elution of compounds released from the solid matrix with a mixture of solvents. As reported in the literature [15,16,17,18,19,20], despite its simplicity, the SSDM process is very effective and non-destructive for many unstable phenolic and non-phenolic compounds. Unfortunately, however, this is not a selective process. As a result of the destruction of the plant tissue, all compounds characteristic for a given matrix are released and eluted with the liquid against the rule “like dissolves like”. This is a disadvantage of the analytical procedure because the obtained extract is characterized by the extraordinary wealth of components, most of which are interfering substances such as chlorophyll, waxes, oils and sterols. These compounds can be deposited in the chromatographic system, changing its properties, therefore they should be removed before the analysis.

SPE is a technique widely applied for the purification of plant extracts, especially with the silica-based octadecyl (C18) sorbent, due to its high sorption capacity and the possibility of fractionating several classes of compounds [9,21,22]. However, the simple C18 SPE procedure is not suitable for acid compounds because of their very small affinity for this reversed-phase (RP) sorbent. There are many approaches in chromatography to alter selectivity and increase the retention of compounds using the RP sorbents. One of them, less known in combination with SPE, is based on the use of reagents capable of creating ion pairs with ionized analyte molecules. The formed ion pairs behave like non-ionic neutral molecules that interact with the non-polar sorbent. This process is called ion-pair solid-phase extraction (IP–SPE) and so far it has been most often used in environmental analysis to determine the initial concentration of phenols, nitrophenols and naphthalene sulfonates from water samples [22,23,24,25]. As follows from the literature, apart from environmental analysis, the IP–SPE process is also used for pharmaceutical analysis [22,26]. However, there is no data in the literature on the use of IP–SPE for extraction of compounds from plant extracts. Therefore, the aim of this study was to develop and optimize a useful method of chlorogenic acid isolation using the SSDM technique with subsequent purification of the obtained extract in the IP–SPE process in order to ensure a reliable analysis of the compound content in plants using the HPLC–DAD technique.

## 2. Results and Discussion

### 2.1. Chromatographic Identification of Chlorogenic Acid

**Figure 1:**
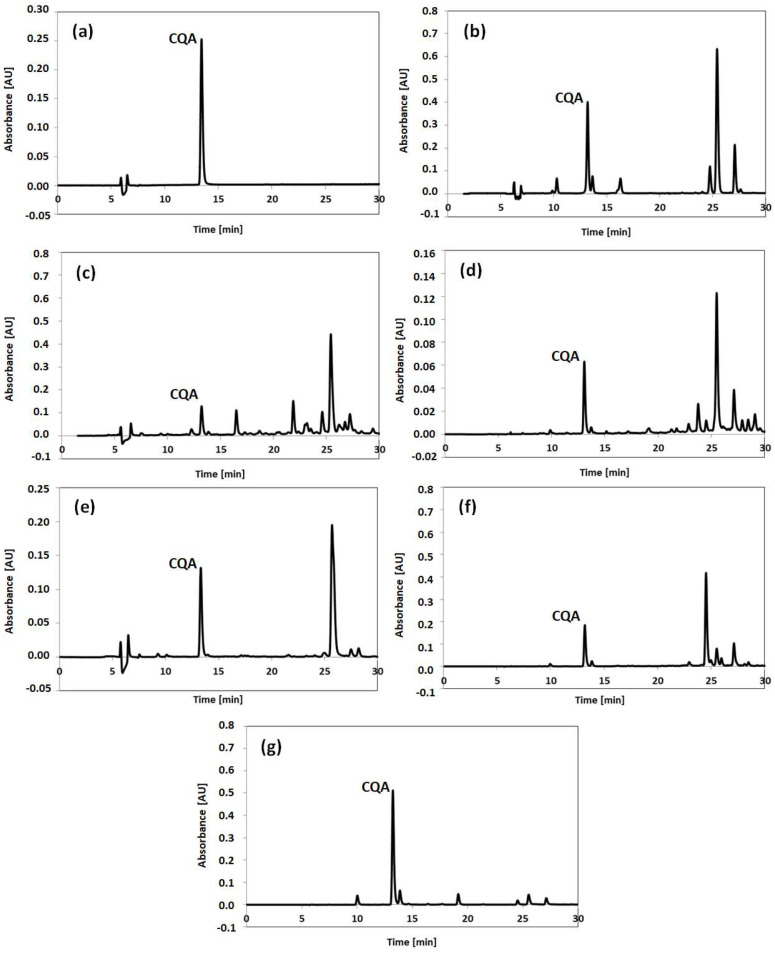
Exemplary chromatograms of the CQA extract after SSDM of the CQA standard (**a**) and water extracts obtained using the SSDM technique from pickled artichoke hearts (**b**), dried chamomile flowers (**c**), tansy (**d**), coltsfoot (**e**), yarrow (**f**) and green coffee beans (**g**).

The IP–SPE process for the purification of CQA was explored using HPLC analysis. Chromatographic separations were performed with the gradient elution profile optimized to obtain a relatively quick analysis with good resolution between compounds peaks in the SSDM extracts of the plants. The exemplary chromatograms of the obtained extracts are shown in Figure 1. The CQA peak was eluted at 13.2 min. The linearity of the method was tested for five concentrations of CQA standard solution at levels from 0.01 µg/mL to 100 µg/mL. The resultant calibration curve (y = 915.84x − 0.4578) was found to be linear in the tested concentration range (R^2^ = 0.9998). The limit of detection (LOD) was calculated using the standard deviation approach by repeated analysis of the standard solution near the targeted LOD. The obtained LOD value was 0.0512 µg/mL.

The chromatogram of the CQA extract after SSDM of the CQA standard is shown in Figure 1a, whereas Figure 1b–g show the chromatograms of SSDM extracts from pickled artichoke hearts, dried chamomile flowers, tansy, coltsfoot, yarrow and green coffee beans, respectively, which were used to demonstrate the practical potential of the developed IP–SPE procedure. Methanol and water were used as the dispersing liquid and as the eluent, respectively, in the applied SSDM procedure. The single peak visible in Figure 1a and derived from the chlorogenic acid standard subjected to the SSDM procedure proves that CQA does not transform or degrade during this process. The well-separated peaks closest to CQA in the chromatograms from Figure 1b–g additionally prove the applicability of the developed HPLC methodology for the quantification of CQA.

### 2.2. Optimisation of the IP–SPE Procedure

To obtain good separation selectivity in IP–SPE, the analyte molecules should be in an ionized form, i.e., at the pH two units above their pKa value. The other parameters influencing separation are the choice of counter-ion type and its concentration. Since tetrabutylammonium (TBA) salts are the most frequently used type of IPR for the analysis of acid compounds [23], tetrabutylammonium bisulfate was selected for the formation of ion pairs with CQA. The influence of TBA on the effectiveness of CQA purification was investigated using the most common RP sorbent, i.e., C18 sorbent. TBA concentration, the pH of the sample, the type of elution solvent mixture, and the process of ion-pair formation (in the liquid phase of the sample or in the sorbent solid phase) were studied as factors affecting the retention and purification efficiency of CQA. The obtained research results are discussed separately below for the factors influencing the efficiency of trapping compounds on the sorbent and the efficiency of their re-extraction from the sorbent. Moreover, given that the SPE process allows not only purification of the extract but also concentration of the desired compound(s), the breakthrough volume is discussed separately as an important parameter for the concentration fold. Finally, based on the results obtained for the CQA standard solution, the optimized IP–SPE process was used to show its practical potential to isolate CQA from aqueous SSDM extracts of plants with proven healing properties.

#### 2.2.1. Effect of TBA Concentration and Sample pH on CQA Trapping

**Figure 2:**
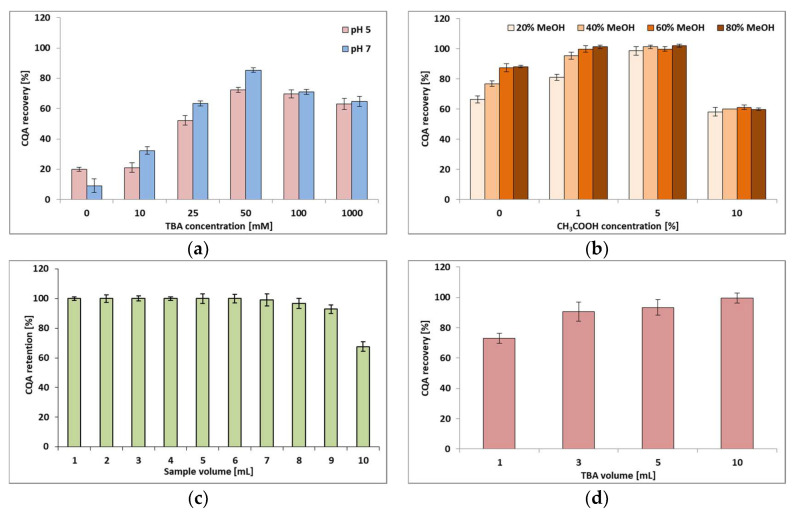
Effects of IP–SPE variables on the recovery(retention) rates of CQA: (**a**) TBA concentrations at two different pH values of chlorogenic acid solution; (**b**) methanol concentrations in the eluting mixture with or without the addition of 1, 5 or 10% *v/v* acetic acid solution; (**c**) volumes of the CQA solution with TBA that was passed through the sorbent during ion-pair formation in the CQA solution; and (**d**) volumes of TBA solution passed through the sorbent during ion-pair formation on the sorbent.

The effect of TBA concentration (10, 25, 50, 100 and 1000 mM) on CQA recovery from the C18 sorbent at two different pH values (5 and 7) of CQA standard solution is shown in Figure 2a. For a better visualization of the studied effect, the figure also contains the CQA recovery obtained without the addition of TBA (bars for 0 mM TBA). In these experiments methanol was used to elute the CQA from the sorbent. The CQA-TBA ion pairs were formed in the CQA solution before loading the sample onto the C18 sorbent. The results of the statistical analysis of the effects of the examined IP–SPE parameters on CQA yield are presented in Table 1.

Analysis of the data presented in Figure 2a reveals that the effectiveness of the IP–SPE process actually depends on the amount of TBA in the solution. The lowest and comparable recoveries of CQA were obtained for the sample without TBA and for the smallest concentration of TBA, i.e., 0.01 M at pH 5. The addition of TBA to the CQA solution improves the affinity of CQA for the sorbent in a statistically significant way (see Table 1). However, the effect depends not only on the amount of TBA in the solution but also on the degree of CQA dissociation. The greatest recovery of CQA is observed at 0.05 M TBA and pH 7, i.e., at the pH value ensuring a higher degree of acid dissociation. If the TBA concentration is greater than 0.05 M, CQA recovery begins to decrease regardless of the pH of the sample. One possible explanation for the observed decline could be the repulsion of the neutral ion pairs by an excess of positively charged TBA ions. It can be assumed that when the concentration of TBA in the solution is large enough and exceeds the amount necessary to form ion pairs with all acid molecules, TBA starts to be preferably adsorbed on the C18 surface. The more TBA is adsorbed on the sorbent surface, the more ion pairs are repelled from the surface and the smaller the acid recovery. Therefore, it can be concluded that the obtained positive surface charge minimizes the retention of neutral ion pairs. Similar conclusions were reached by researchers investigating the enrichment of peptides using IP–SPE in [26].

#### 2.2.2. Optimization of Elution Conditions

To establish the optimum conditions for the elution of CQA from the C18 sorbent, different compositions of methanol–water mixture were tested. As is known, in the case of phenolics chromatographed in reversed-phase mode, their elution is enhanced by the presence of hydrogen ions in the system’s mobile phase. Therefore, the influence of methanol content in the acidified aqueous solution was also examined. As the mobile phase of the applied HPLC system was composed of an aqueous solution of acetic acid, this organic acid was chosen for the experiments, at concentrations of 1, 5 and 10% *v/v* CH_3_COOH in water. The results obtained are presented in Figure 2b. Their statistical analysis is summarized in Table 1.

These data were obtained for the TBA concentration with the best results in the previous series of experiments, i.e., for 0.05 M of TBA with the sample pH adjusted to 7.0. Although Figure 2b contains only the data obtained for the elution step, i.e., the final step of the SPE procedure, it should be stressed that for complete control of the process, the eluates obtained during the sorbent loading and washing step were also analysed. Analysis of these samples did not reveal the presence of any analyte.

Analysing the data summarized in Figure 2b, it can be generally stated that the change in concentration of both methanol and acetic acid in the eluent changed the efficiency of CQA re-extraction from the sorbent. The greatest changes were observed for the aqueous methanol solution and the methanol solution in 1% acetic acid. The validity of this conclusion is confirmed by the Fischer coefficient values from Table 1 (79.71 and 63.93 respectively for *F_tab_* = 4.07). Increasing the acid concentration to 5 and 10% neutralized the effect of the increase in elution strength resulting from increasing the concentration of methanol in the eluent, as indicated by the fact that the changes in the CQA recovery were statistically insignificant for both concentrations (*F_cal_* < *F_tab_*). Nevertheless, at the highest concentration of acetic acid, i.e., 10%, an increase in the retention behaviour of CQA on the sorbent was observed and the re-extraction efficiency was lowest. In view of the above, a 20% methanol solution in 5% acetic acid was considered to be the optimal mixture for eluting CQA from the sorbent, allowing control of the selectivity of the IP–SPE process in the re-extraction step. This mixture was used in the next series of experiments.

#### 2.2.3. Determination of Breakthrough Volume

Breakthrough volume is one of the parameters of the SPE process frequently used for determination of the strength of analyte interaction with a given mass of sorbent. When the breakthrough volume is exceeded, the analyte begins to elute from the sorbent in the sample loading stage, decreasing the extraction efficiency. In order to determine the volume of sample that can be passed through the sorbent without CQA loss, a series of SPE tubes packed with the same mass of C18 sorbent (500 mg) were loaded with increasing volumes of CQA solution with TBA, and the eluate was subjected to HPLC analysis. As with previous stages in this series of experiments, the solution obtained after mixing 2 mL of 0.05 M TBA solution with 18 mL of aqueous CQA solution (pH 7.0) was used. The holding volume of this mass of sorbent was approximately 0.5 mL. The obtained results are given in Figure 2c.

As follows from Figure 2c, after passing 8 mL of the above-described mixture through the sorbent, CQA recovery was approximately 97%. Adding another portion of the sample reduced acid recovery to 93%. Therefore, it can be assumed that the sample volume that can be loaded onto the sorbent without analyte loss is 8 mL, which indicates a sorption capacity of 1.6 mg CQA.

#### 2.2.4. Comparison of the Efficiency of Ion-Pair Formation in the Water Phase and in the Solid Phase

The data presented above were obtained under conditions such that the ion pairs between TBA+ and dissociated chlorogenic acid molecules were formed in the liquid phase. The ion pairs can be formed in a dynamic way on the sorbent surface as well. In this case, the sorbent surface becomes saturated with TBA ions sooner. To compare the effect of the ion-pair formation mode (in the liquid or solid phase) on the efficiency of CQA extraction, different volumes of TBA solution containing the same amount of TBA were passed through the previously conditioned C18 material. Then, 1 mL of the CQA solution adjusted to pH 7 was loaded onto the sorbent. Finally, CQA was eluted with 10 mL of a mixture of methanol in a 5% aqueous solution of acetic acid (20:80% *v/v*). The results are presented in Figure 2d and Table 1.

As can be seen from the presented data, although in each case the same amount of TBA was loaded onto the C18 material, CQA recovery differed depending on the volume of TBA solution passed through the sorbent (*F_cal_* = 17.80, *F_tab_* = 4.07). While a volume of 1 mL was not sufficient for the effective retention of acid ions on the sorbent, the volume of 10 mL was large enough. It is clear that a volume of 10 mL guarantees a uniform charge distribution in the applied bulk of the solid phase, giving an almost 100% recovery of CQA. As follows from the presented data, the method of ion pair formation does not affect CQA recovery under optimized conditions. However, it should be stressed that the formation of ion pairs in a CQA solution is easier in execution and reduces the time needed for the IP–SPE procedure. Therefore, the method of creating ion pairs was applied in the further research stage.

### 2.3. Application of IP–SPE Procedure for Estimation of CQA Amount in the Plant Extracts

To demonstrate that the IP–SPE procedure optimized in the model system using a CQA standard solution works in real systems with real plant extracts, several plants were selected for testing. SSDM extraction was performed under optimized conditions [14] using water as the SSDM eluent. The conditions of the IP–SPE procedure considered optimal for the CQA standard solution were adapted for these plant extracts. The obtained results are summarized in Table 2. Their analysis, supported by data in the literature [1,9,13,14] on CQA content, allows us to confirm that the developed methodology is a promising tool to control the selectivity of the SSDM method, making this extremely simple and non-destructive technique for CQA isolation even better.

## 3. Materials and Methods

### 3.1. Plant Materials and Chemicals

Dried flowers of tansy (*Tanacetum vulgare* L.), chamomile (*Matricaria chamomilla* L.), coltsfoot (*Tussilago farfara* L.) and yarrow (*Achillea millefolium*) along with green coffee beans and pickled artichoke hearts were purchased at a local market (Lublin, Poland). Sufficiently large representative portions of each plant material (approx. 250 g) were ground, precisely weighed and subjected to the SSDM procedure.

Methanol (analytical reagent-grade), acetonitrile (HPLC-grade), 80% acetic acid (analytical reagent-grade), NaOH, *n*-hexane (analytical reagent-grade), sodium phosphate and phosphoric acid were purchased from the Polish Chemicals Plant POCh (Gliwice, Poland). Water was purified using the Milli-Q system from Millipore (Millipore, Bedford, MA, USA). The sand used as the abrasive material in SSDM was contributed by the local glassworks. It was fractionated, leached with 1 M HCl, washed out with distilled water to neutrality and dried. The 0.2–0.4 mm fraction was applied in the experiments. Octadecyl columns prepared in-house by packing 6-mL polypropylene SPE tubes with 500 mg of Supelclean LC-18 material obtained from Supelco Park (Bellafonte, PA, USA) were employed for SPE. The standard of chlorogenic acid (1,3,4,5-tetrahydroxycyclohexane carboxylic acid 3-(3,4-dihydroxycinna-mate)) was purchased from Merck (Darmstadt, Germany). Tetrabutylammonium hydrogen sulfate (TBA) was purchased from Sigma-Aldrich (Poznań, Poland).

### 3.2. Sea Sand Disruption Method

SSDM was performed according to the validated procedure described elsewhere [14,16,19]. Briefly, the pre-ground sample (0.2 g) was placed in the glass mortar and mixed with the appropriate excess of sand (1:4). After adding 1.0 mL of methanol, used as the dispersant liquid, the mixture was ground for 10 min with a glass pestle to obtain a homogeneous mixture, the so-called SSDM blend. During grinding, the sides of the mortar and the pestle were scraped occasionally with a spatula to ensure the best possible homogenization. After homogenization, the SSDM mixture was quantitatively transferred to a 5 mL syringe barrel lined with a paper frit. The blend was pressed in the barrel with a syringe plunger and then eluted with portions of water into a 25 mL calibrated flask. For statistical purposes, the SSDM procedure for the given plant material was repeated three times.

### 3.3. IP–SPE Procedure

The IP–SPE process was conducted using a Supelco 12-port vacuum manifold with C18 sorbent (500 mg) packed into 6-mL SPE tubes. The optimized IP–SPE procedure that was applied involved the following steps:

1. The sorbent was pre-conditioned by passing 5 mL of methanol followed by 5 mL of deionized water.
2. The tube was loaded with 1 mL of the sample obtained by mixing the plant SSDM extract with 50 mM TBA solution in a 9:1 volume ratio. The sample pH was adjusted to 7.0.
3. The column was washed with 5 mL of deionized water.
4. The analyte was eluted into a 10 mL volumetric flask using a solution of 20% methanol in 5% acetic acid. The resulting extract was analysed by HPLC.

The C18 material was exposed to relatively high and low concentrations of hydrogen ions. However, no sorbent degradation was observed. Therefore, the sorbent was used many times. In order to prepare the material for re-use, the sorbent was regenerated under the following conditions: washing with 5 mL of acetone, drying under vacuum, washing with 5 mL of *n*-hexane and drying again. When the regeneration procedure was carried out, the sorbent could be reconditioned with methanol.

To determine the optimal IP–SPE conditions for the quantification of CQA in the extracts, the influence of the following parameters on the CQA recovery was investigated:

1. TBA concentration (0, 10, 25, 50, 100 or 1000 mM) with the other IP–SPE parameters: volume of TBA solution, 1 mL; volume of CQA standard solution (or plant extract sample), 9 mL; pH of standard solution (or plant extracts) adjusted to the value of 5 or 7; volume of eluent methanol, 10 mL;
2. Methanol concentration in the elution mixture (20, 40, 60 or 80% *v/v*) without or with the addition of acetic acid solution at concentrations of 1, 5 or 10% *v/v*; with the other IP–SPE parameters: volume of TBA solution, 1 mL, concentration of the TBA solution, 50 mM; volume of CQA standard solution (or plants extract samples), 9 mL; pH of the standard solution (or plant extracts) adjusted to 7; eluent volume, 10 mL;
3. Volume of CQA solution with TBA (ranging from 1 to 10 mL) with the other IP–SPE parameters: ratio of CQA volume to TBA, 9:1; TBA concentration, 50 mM; pH of standard solution adjusted to 7.

In the course of optimization, the filtrate, washing solvent and eluates were collected into 10 mL volumetric flasks, filled up to this volume with deionized water, where necessary, and analysed by HPLC. For statistical purposes, each IP–SPE procedure was repeated five times.

The experiments also investigated the effect of the way in which ion pairs formed with the CQA and TBA. Apart from the formation of ion pairs in a solution (the above experiments) which was then subjected to the SPE procedure, the ion pairs were also formed dynamically during the passage of the extract through the C18 sorbent after its initial saturation with the IP reagent. In the latter case, different volumes of the TBA solution containing the same amount of TBA were passed through the C18 sorbent (500 mg). Then 1 mL of CQA solution or plant extract was applied to the prepared sorbent. The TBA solutions used in this series of experiments were obtained from a 25 mM TBA solution by diluting 0.2, 0.6, 1.0 or 2.0 mL of the solution in 1, 3, 5 and 10 mL of water, respectively. In all experiments, the flow of liquid through the sorbent was constant and equal to 1 mL/min. The CQA standard solution used in all optimization experiments of the IP–SPE procedure was obtained by dissolving 10 mg of chlorogenic acid standard in 50 mL of water. This amount of CQA is roughly equal to that characteristic of the examined plant extracts.

### 3.4. HPLC Analysis

The HPLC measurements were performed using a Dionex liquid chromatograph (Dionex Corp., Sunnyvale, CA, USA) composed of a chromatography enclosure (LC20) containing a PEEK automated injection valve with a 25 µL sample loop, a gradient pump (GP50) and a photodiode array detector (PDA100). The whole chromatographic system was under the control of the PeakNet6 data acquisition system. Chromatographic separations were made at 25 °C using the Prodigy ODS-2 column (5 µm, 250 × 4.6 mm I.D.) (Phenomenex, Torrance, CA, USA) and the guard column of the same material. The mobile phase components were as follows: acetic acid–water mixture containing 0.5 mL of 80% acetic acid in 100 mL of solution (solvent A), and acetonitrile (solvent B). The analyses were performed in a linear gradient of solvent B from 5 to 40 mL B/100 mL (0–46 min). At the end, the percentage of B was decreased to the initial value of 5 mL B/100 mL for 2 min, and before the next analysis, the column was equilibrated using a mobile phase containing 5 mL B/100 mL for 15 min. The mobile phase flow rate was 0.5 mL/min. In the course of each run, absorbance spectra in the range 200–600 nm were collected continuously. CQA was monitored at 270 and 330 nm. The quantification of CQA in the analysed samples was based on its calibration curve. The qualification was made by comparing the CQA UV spectrum (data not presented) with those for the reference standard and data from the literature.

### 3.5. Statistical Analysis

All data are expressed as mean ± standard deviation. Analysis of variance (ANOVA) and F-tests were used to assess the influence of experimental factors on the CQA yield. If the calculated value of *F* (*F_cal_*) exceeds the table value, *F* (*F_tab_*), this indicates a statistically significant influence of the given parameter. The values were considered to be significantly different when the result of the compared parameters differed at a significance level of *p* = 0.05. To determine the significance of each Fisher coefficient, *p*-values were used.

## 4. Conclusions

Comprehensive evidence of the health-promoting effects of chlorogenic acid has resulted in a significant increase in interest in this compound among scientists and average consumers observing a healthy diet. As follows from the literature [9,10,11,12,13], CQA is an unstable compound. When exposed to high temperatures it easily degrades to other substances that may be mistakenly interpreted as natural plant components. Therefore, reliable analysis of the CQA content requires an appropriate method of isolation from the plant material. The research results presented in [14] revealed that SSDM is a non-destructive method of efficient extraction. However, this is a non-selective method because as a result of grinding the plant tissue with sand, all compounds are released and eluted with the liquid against the rule of thumb “like dissolves like”. These compounds can be deposited in a chromatographic system, changing its properties, including those related to separation. In order to increase the selectivity of CQA isolation from the aqueous extracts obtained via the SSDM method and to eliminate non-acidic interfering substances capable of interacting with the C18 sorbent, in this study an IP–SPE procedure was developed and optimized using TBA as a reagent for creating an ion pair with CQA. Based on the analysis of the CQA standard solution, it was shown that the developed methodology provides reproducible recovery of the analyte (>98%). To verify its practical potential, it was used to isolate CQA from various plants. By comparing the obtained CQA contents with those available in the literature, the high analytical capabilities of the proposed procedure were demonstrated.

## Figures and Tables

**Table 1:** *F*-Values and *p*-Values obtained from the variance analysis of the experimental results.

Effects	*F_cal_*-Value	*p*-Value	*F_tab_*-Value
^1^ Main effect of sample pH	25.92	3.30 × 10^−05^	4.26
^1^ Main effect of TBA concentration	535.78	8.60 × 10^−24^	2.62
^1^ Interaction effect	16.91	3.51 × 10^−07^	2.62
^2^ Effect of MeOH concentration without acetic acid	79.41	2.71 × 10^−06^	4.07
^2^ Effect of MeOH concentration with acetic acid (1%)	63.93	6.22 × 10^−06^	4.07
^2^ Effect of MeOH concentration with acetic acid (5%)	2.26	0.16	4.07
^2^ Effect of MeOH concentration with acetic acid (10%)	1.69	0.24	4.07
^1^ Main effect of MeOH concentration	1132.22	1.52 × 10^−32^	2.90
^1^ Main effect of acetic acid concentration	99.22	2.74 × 10^−16^	2.90
^1^ Interaction effect	22.65	1.81 × 10^−11^	2.19
^2^ Effect of TBA volume	17.80	6.71 × 10^−04^	4.07

^1^ Two-way ANOVA with repetition. ^2^ One-way ANOVA.

**Table 2:** Amount [mg/g] of chlorogenic acid estimated in the plants by the SSDM method in combination with the IP–SPE procedure (mean value ± SD).

Amount of Chlorogenic Acid Estimated in					
Artichoke	Chamomile	Tansy	Coltsfoot	Yarrow	Green Coffee Beans
26.32 ± 1.31	2.13 ± 0.09	6.48 ± 0.61	5.54 ± 0.89	7.02 ± 0.32	48.96 ± 2.25

## Sample Availability

Not available.
